# Correlation of diffusion tensor imaging parameters with neural status in Pott’s spine

**DOI:** 10.1051/sicotj/2016014

**Published:** 2016-04-29

**Authors:** Nikhil Jain, Namita Singh Saini, Sudhir Kumar, Mukunth Rajagopalan, Kanti Lal Chakraborti, Anil Kumar Jain

**Affiliations:** 1 University College of Medical Sciences & GTB Hospital 110095 Delhi India; 2 Department of Radiology, Institute of Nuclear Medicine and Allied Sciences (INMAS) 110054 Delhi India; 3 Sharda University 201306 Greater Noida India

**Keywords:** Diffusion tensor imaging, DTI, Spinal tuberculosis, Pott’s spine

## Abstract

*Introduction*: Diffusion tensor imaging (DTI) has been used in cervical trauma and spondylotic myelopathy, and it has been found to correlate with neural deficit and prognosticate neural recovery. Such a correlation has not been studied in Pott’s spine with paraplegia. Hence, this prospective study has been used to find correlation of DTI parameters with neural deficit in these patients.

*Methods*: Thirty-four patients of spinal TB were enrolled and DTI was performed before the start of treatment and after six months. Fractional anisotropy (FA), Mean diffusivity (MD), and Tractography were studied. Neurological deficit was graded by the Jain and Sinha scoring. Changes in FA and MD at and below the site of lesion (SOL) were compared to above the SOL (control) using the unpaired *t*-test. Pre-treatment and post-treatment values were also compared using the paired *t*-test. Correlation of DTI parameters with neurological score was done by Pearson’s correlation. Subjective assessment of Tractography images was done.

*Results*: Mean average FA was not significantly decreased at the SOL in patients with paraplegia as compared to control. After six months of treatment, a significant decrease (*p* = 0.02) in mean average FA at the SOL compared to pre-treatment was seen. Moderate positive correlation (*r* = 0.49) between mean average FA and neural score after six months of treatment was found. Tractography images were not consistent with severity of paraplegia.

*Conclusion*: Unlike spondylotic myelopathy and trauma, epidural collection and its organized inflammatory tissue in Pott’s spine precludes accurate assessment of diffusion characteristics of the compressed cord.

## Introduction

Neurological complications in spinal tuberculosis result from extrinsic compression on the spinal cord by an abscess, granulation tissue, caseous material, tubercular debris, and/or mechanical instability of the vertebral column. The intrinsic changes in spinal cord include cord edema, myelomalacia, cord atrophy, direct involvement of meninges or cord by infection, and infrequently infective thrombosis or endarteritis of spinal vessels [1].

Myelomalacia, cord atrophy, and syringomyelia of the spinal cord are indicators of poor neural recovery while preserved spinal cord volume, edema/myelitis, and wet lesion on MRI usually show good neural recovery [1]. A relationship between residual cord size, cerebrospinal fluid (CSF) anterior to cord, signal intensity changes on T2 images, and neural deficit has been shown [2]. In spite of these correlations, it is not possible to prognosticate neural improvement.

**Figure 1.**
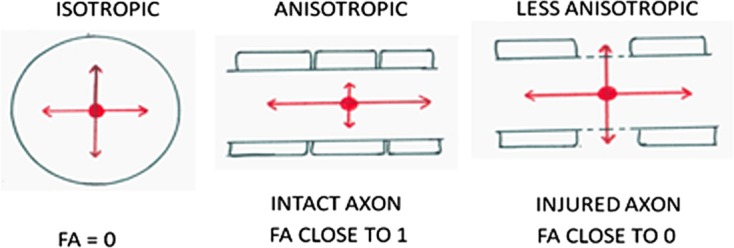
Isotropic and anisotropic diffusion of water molecules.

Diffusion tensor imaging (DTI) measures magnitude and direction of diffusion of water molecules due to their inherent latent thermal energy. Restriction of diffusion of water molecules by biological barriers (myelin sheath of axons of white matter) results in diffusion in one particular direction, known as anisotropic diffusion (range 0–1). The parameter measuring this anisotropy is fractional anisotropy (FA) (Figure 1). The overall magnitude of diffusion is measured by apparent diffusion coefficient (ADC)/mean diffusivity (MD). Intact neurons have a FA value close to one. With disruption, FA value decreases (less anisotropic) and MD value increases. Visual representation of diffusion can be shown by color-coded tracts, this being known as tractography [3].

DTI is reported to be useful in cervical spondylotic myelopathy and trauma [4–21]. DTI parameters have been correlated with neurological deficits, mainly motor score [4, 7, 10, 12, 16, 18, 20], and have shown to predict neural outcome in few studies [6, 10, 13].

The correlation of DTI observations with neural deficit has not been described in spinal tuberculosis. If such a correlation can be found then it may be possible to prognosticate the neural recovery. Hence we present this study to evaluate the DTI observations in these patients and find a correlation with neural status.

## Materials and methods

Thirty-four consecutive patients of spinal tuberculosis (C3-D12 vertebral body) were enrolled for the study after prior approval from appropriate Ethics Committee and informed consent from patients. Patients were divided into two groups based on neurological deficit, group A – without neurological deficit (*n* = 16) and group B – with neurological deficit (*n* = 18). Diagnosis was made on clinical and imaging features and by histopathology in operated patients. Patients who had concomitant spinal pathology/chronic illness, having metallic implants in situ, or claustrophobia were excluded from the study. Neurological assessment was done by the Jain and Sinha scoring [22]. DTI was performed in all patients before the start of treatment and after six months of treatment.

*DTI Protocol*: DTI was performed at the Institute of Nuclear Medicine and Allied Sciences (INMAS) on 3 Tesla Siemens Magnetom Skyra (syngo MR D11) system. A total of 25 axial sections at 5 mm thickness were obtained to cover one vertebral segment above and one below the involved segment of the spinal cord with a distance factor = 2 mm; field of view (FOV) = 280 × 280 mm; repetition time (RT) = 4100 ms; echo time (TE) = 66 ms; NEX = 4; fat suppression = Spair; Matrix = 128 × 128; diffusion directions = 20; *b* values = 0 and 700. The DTI imaging plane was parallel to the conventional axial images; perpendicular to the long axis of the spinal cord.

**Figure 2.**
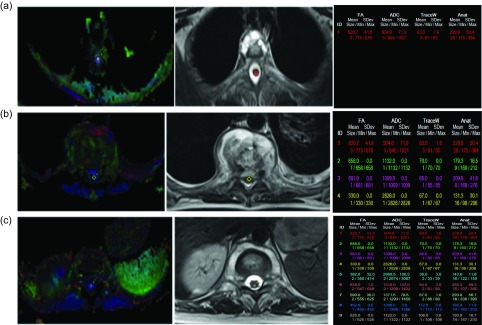
(a) Left: circular ROI drawn for calculation of FA and MD/ADC values on diffusion FA map above the site of lesion (SOL). Middle: corresponding T2 axial MRI image shows no disease above the SOL. Right: once circular ROIs were drawn, FA and MD values were generated which were recorded. Three such ROIs were drawn above the SOL. (b) Left: circular ROI drawn at the SOL. Middle: corresponding axial MRI image shows disease and compression on cord. Right: FA and MD values generated in the same color as ROI. Note: The first three values are for above the SOL. (c) Left: circular ROI drawn below the SOL. Middle: the corresponding axial MRI image shows no disease. Right: FA and MD values generated. Values (1–3) – above SOL (4–6) – at SOL (7–9) – below SOL.

*Image Processing*: Quantitative analysis of DTI data was performed using software on a Syngofast Viewer imaging software platform (Siemens Magnetom Skyra). Maps of FA and MD were generated with background noise suppressed by using the same software. The regions of interest (ROI) were carefully placed on the spinal cord so that they included both central gray and white matter with standard deviation ≤10%. ROI locations were confirmed by using the optimal conventional MRI sequence, and care was taken to avoid inclusion of CSF. Spinal cord FA and MD values were calculated from the DTI metrics on a voxel-by-voxel basis and displayed as two-dimensional color and grayscale images by manually drawn ROIs and averaging three ROI values in each of the segments evaluated (above, at, and below the lesion). The size of ROIs had to be adapted to the axial size of the spinal cord (Figure 2).

*Control group*: The values of the unaffected cord above the site of lesion (SOL) were taken as control as these values reflect the normal parameters for that patient against which values at and below the SOL were compared.

The following parameters were evaluated:

- *Fractional anisotropy* (*FA*): The FA values were calculated at and below the SOL (Figure 3). The mean FA values at and below the SOL of group A were compared to the values of group B. In both groups the mean FA values at and below SOL were compared to control. In both groups, the mean FA values at and below the SOL before the start of treatment were compared to the values of the same sites after six months. The correlation of the mean FA value at the SOL to the severity of paraplegia, both before the start of treatment and after six months, was also done.
  **Figure 3.**
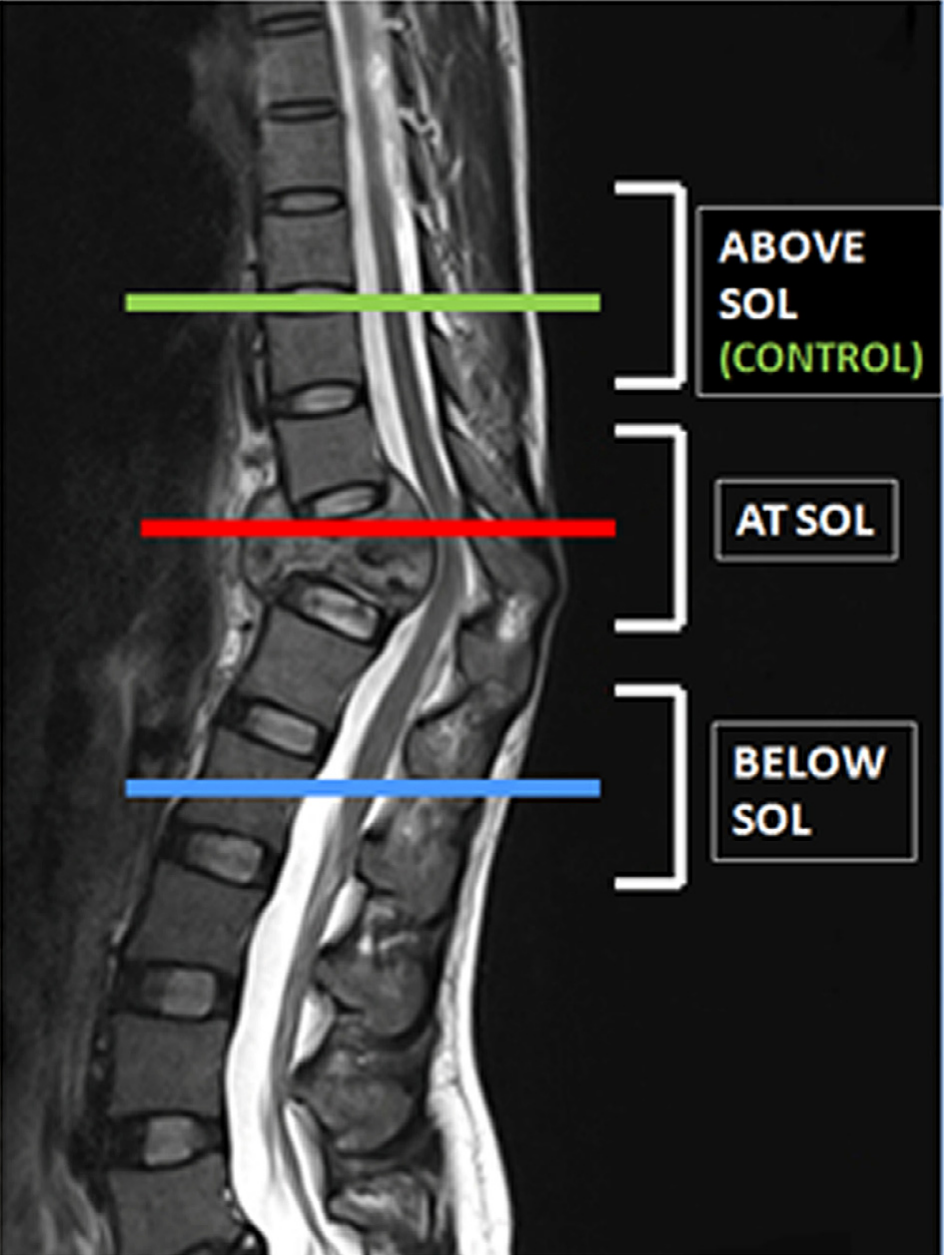
Levels of site of lesions (SOL) as marked on a T2 sagittal MRI image.
- *Mean diffusion* (*MD*): The same comparisons were done for MD values as well, at the start of treatment and after six months.
- *Fiber tractography*: The visual impression of fiber tract volume was analyzed with respect to severity of paraplegia.

*Treatment protocol*: All patients were treated initially with bed rest, category 1 DOTS antitubercular therapy (ATT), and suitable braces. Patients who had severe paraplegia, unstable spine, or their neural status worsened on treatment were operated. Antero-lateral decompression (ALD) with bone grafting (with/without posterior instrumented stabilization) was the procedure performed.

*Statistical analysis*: The data collected was entered in SPSS 17.0 for analysis. Changes in FA and MD were recorded on the scale of 0–1 and 0–3, respectively. The t-test was used to study the values at and below SOL against control. In each group, values before and after treatment were compared using the paired *t*-test. FA and MD at the SOL were correlated to the severity of paraplegia by the Pearson coefficient. *P* value less than 0.05 was considered as statistically significant.

## Results

The mean age of patients was 31.2 years (range 15–70 years) with 12 male and 22 females. In group B, four patients had grade 2, while three and 11 patients had grade 3 and grade 4 paraplegia, respectively. Two patients were lost to follow-up. The calculation of DTI parameters on follow-up was not possible in six patients due to post-operative implant artifacts.

- Fractional anisotropy (Table 1)
  **Table 1.** Mean FA values in groups A and B.

Mean FA value	Before treatment			After 6 months treatment		
	Above the SOL	At the SOL	Below the SOL	Above the SOL	At the SOL	Below the SOL
	(Mean ± *SD*)	(Mean ± *SD*)	(Mean ± *SD*)	(Mean ± *SD*)	(Mean ± *SD*)	(Mean ± *SD*)
Group A	0656 ± 0.08	0.604 ± 0.10	0.548 ± 0.11	0.592 ± 0.09	0.552 ± 0.11	0.558 ± 0.11
Group B	0.632 ± 0.12	0.618 ± 0.12	0.528 ± 0.12	0.605 ± 0.11	0.536 ± 0.08	0.559 ± 0.15
*Pre-treatment*: The value at the SOL was not statistically different from control (both groups).
  In group A, the mean FA value was *significantly lower* (*p* = 0.01) below the site of lesion (SOL) as compared to control. In group B also, the mean FA value was *significantly lower* (*p* = 0.001) below the SOL when compared to control.
*After six months*: In group A, the difference between mean average FA (at and below SOL) values before treatment and after six months was not significant.
  In group B, there was a *significant decrease* (*p* = 0.02) in FA after six months of treatment at the SOL as compared to pre-treatment.
- Mean diffusivity (Table 2)
  **Table 2.** Mean MD values in groups A and B.

Mean MD value	Before treatment			After 6 months treatment		
	Above the SOL	At the SOL	Below the SOL	Above the SOL	At the SOL	Below the SOL
	(Mean ± *SD*)	(Mean ± *SD*)	(Mean ± *SD*)	(Mean ± *SD*)	(Mean ± *SD*)	(Mean ± *SD*)
Group A	1.268 ± 0.20	1.277 ± 0.29	1.240 ± 0.16	1.410 ± 0.27	1.412 ± 0.39	1.249 ± 0.25
Group B	1.211 ± 0.35	1.191 ± 0.42	1.212 ± 0.26	1.205 ± 0.24	1.277 ± 0.26	1.045 ± 0.21
*Pre-treatment*: In groups A and B, the mean MD values at and below the site of lesion (SOL) were not significantly different from control.
*After six months*: In group A, the mean MD values as compared to pre-treatment values had insignificant difference at and below SOL. In group B, after six months the mean MD value showed a *significant decrease* (*p* = 0.04) below the SOL as compared to pre-treatment.
- Correlation with neural status:
  The mean FA value showed moderate positive correlation (*r* = 0.49) with motor score after six months of treatment. Rest of the correlations of pre-treatment FA and MD with sensory and motor score were insignificant.
- Tractography (Table 3 and Figure 4)
  **Figure 4.**
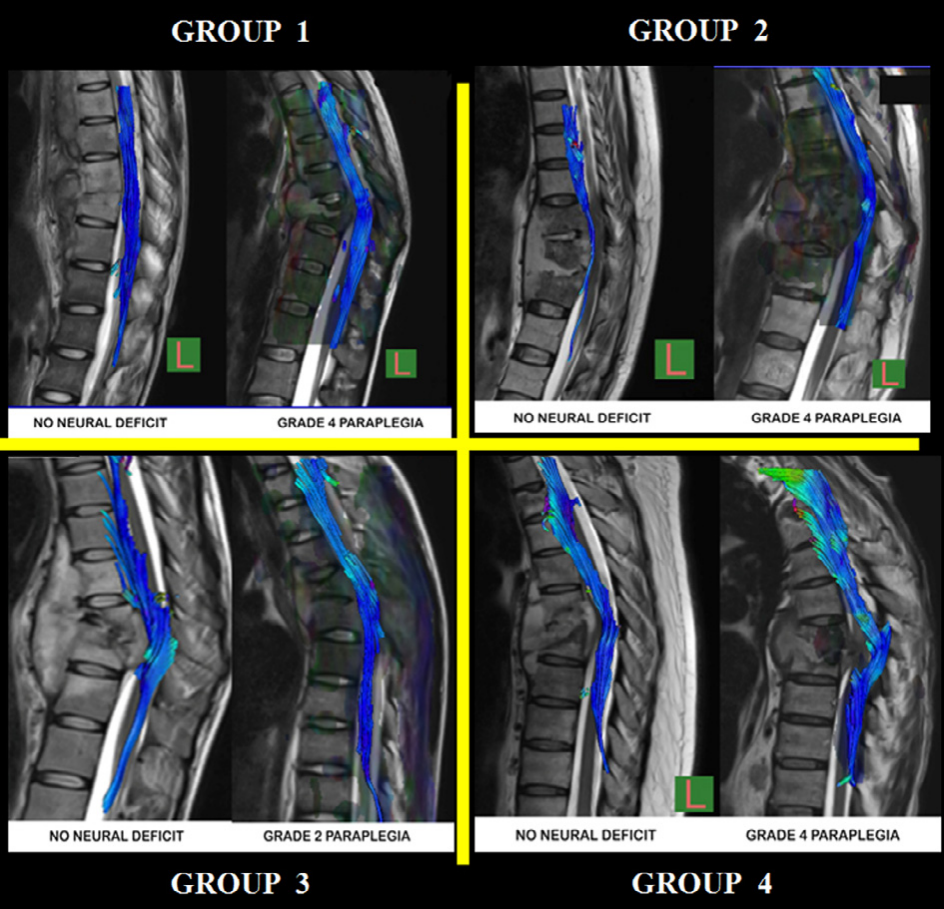
Three-dimensional tractography image acquired in the medio-lateral plane superimposed on sagittal T2WI images shown for each group. Subjective assessment of tract volume at and below site of lesion (SOL) as compared to above SOL shows that in each group, similar images are seen among patients without and with neural deficit.
  **Table 3.** Results of tractography among groups A and B.

	Group A (Number of patients)		Group B (Number of patients)	
	Before treatment	After 6 months (compared to before treatment)	Before treatment	After 6 months (compared to before treatment)
Group 1	6/16	Same – 4/6 Increased – 1/6 No proper tracts* – 1/6	7/18	Same – 2/7 Decreased – 1/7 No proper tracts# – 4/7
Group 2	4/16	Same – 1/4 Increased – 2/4 No proper tracts* – 1/4	3/18	Same – 1/3 Increased – 1/3 No proper tracts# – 1/3
Group 3	2/16	Same – 1/2 Increased – 1/2	4/18	Same – 2/4 Increased – 1/4 Decreased – 1/4
Group 4	3/16	Increased – 1/3 Decreased – 1/3 No proper tracts* – 1/3	3/18	Increased – 1/3 Decreased – 1/3 No proper tracts# – 1/3
Group 5*	1/16			

* Proper tracts could not be drawn.

# Post-operative implant artifacts.

In both groups A and B, volume of tracts was analyzed in the following five groups (as compared to the above SOL):

Group 1 – same volume of tracts at and below SOL;
Group 2 – reduced volume of tracts at and below SOL;
Group 3 – same volume of tracts at SOL and decreased volume below SOL;
Group 4 – decreased volume of tracts at SOL and same volume below SOL;
Group 5 – proper tracts could not be drawn.

## Discussion

The study of DTI of the brain shows that high anisotropy is maintained by organized membranes, and in the spinal cord by myelin sheath and axonal structures. A decrease in FA results from mechanical disruption, tearing, wallerian degeneration, and demyelination.

The FA values, as we move cranial to caudal in the normal spinal cord, decrease (range 0.750–0.570) from cervical to lumbar cord. This decreasing trend of FA from the cervical to lumbar cord is explained by the decrease in white matter to gray matter ratio from cervical to lumbar region. Gray matter percentage is maximum in the lumbar cord (36%), 18% in cervical, and 13.2% in the thoracic cord [23]. In group A of this study, there was significant decrease of FA below the SOL (*p* = 0.01) as compared to control. There were 11/16 patients in group A having dorso-lumbar (DL) disease, therefore the values below the SOL would be predictably low due to the aforementioned reason.

**Figure 5.**
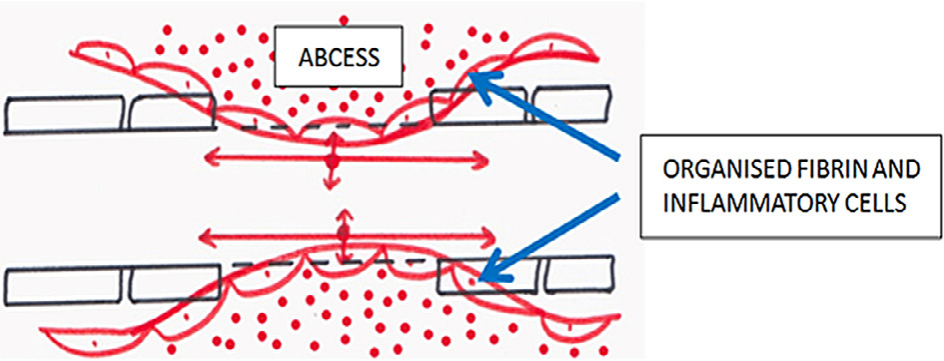
Effect of abscess and organized inflammatory cells on diffusion, thereby maintaining anisotropy.

In group B, the mean FA was lower at the SOL (statistically insignificant) and significantly (*p* = 0.001) lower below the SOL when compared to control. In spite of compression, the FA was not statistically lower at the SOL, as was expected. In cervical spondylotic myelopathy, FA has been found to be significantly lower at the level of compression when compared to controls [6–13]. However, whether the nature of compression (non-infective vs. infective) affects the diffusion characteristics of the cord differently has not been reported to date. The diffusion characteristics of an infective lesion have been studied by Gupta et al. [24] in eight patients with a brain abscess. They found a significantly increased FA and decreased MD in the abscess wall as compared to the cavity. The presence of inflammatory cells and organized fibrin in these abscess walls does not allow free water diffusion, rather water flows along the cell membrane of these cells and fibrin tissue thus maintaining anisotropy. Similarly, in patients with spinal cord compression and significant epidural collection, the inclusion of an epidural collection while drawing the region of interest (ROI) may be responsible for such findings in the present study (Figure 5). A previous study from the author’s institution observed similar findings, however follow-up and correlation with neurological score were not done [25].

The significantly (*p* = 0.001) lower mean FA below the SOL is probably due to distal wallerian degeneration of the cord. Peterson et al. [4] also found that in long-standing injury there is progressive axonal degeneration and dieback which leads to cord atrophy at regions remote from injury and has significantly lower FA values. In chronic compression of the cord, there is axonal restructuring and distal degeneration of the cord [21].

Twenty-six out of 34 patients were followed up by repeat DTI at six months of treatment. DTI was not possible to perform in one patient of group A and in five patients of group B due to post-operative implant artifacts. Two patients of group B were lost to follow-up. Follow-up DTI could not be done due to post-operative implant fixation as mentioned by Kara et al. [9] and Peterson et al. [4].

In group B, there was a significant (*p* = 0.02) decrease in FA at the SOL six months post-treatment as compared to control. As previously mentioned, the presence of inflammatory cells and organized fibrin due to epidural spread could lead to a false high FA. After six months of treatment, epidural collection had resolved in 7/11 patients who were followed up, therefore removing the influence of an epidural collection. That is why a significantly lower FA value at the SOL was observed post six months of treatment which could in fact reflect actual cord diffusion characteristics.

After six months of treatment, a moderate positive correlation (*r* = 0.49) between the motor scores and mean FA was seen. That is, higher mean FA scores at follow-up were likely to have higher motor scores. This kind of correlation between FA and motor scores has been observed in various studies [4, 7, 10, 12, 16, 18, 20]. As the pre-treatment values were not truly representative due to the epidural collection, a significant correlation with motor score was not found. After six months of treatment and resolution of the epidural abscess in the majority of patients, the mean average FA could have been more accurate and hence a correlation was seen. There was no significant correlation between FA and sensory scores or MD with motor or sensory scores.

Tractography was not found to be useful as the visual impression of tracts was not indicative of severity of the neural deficit and reduced tract volumes were found in patients of both groups. The presence or absence of an epidural collection also did not have a uniform effect on tractography. This is in contrast to some studies in which tractography has been shown to delineate transected tracts in cases of traumatic hemisection of the spinal cord [15, 17]. Chang et al. [5] have also found tractography to correlate with motor score in cervical myelopathy.

## Conclusion

The epidural collection in patients of Pott’s spine resulted in false higher FA values. In patients with no epidural collection at follow-up, the values seem to be more accurate and correlate with neural deficit. Mean FA had moderate positive correlation with motor score at six months follow-up. There was lower FA below the site of lesion in paraplegics reflecting wallerian degeneration. DTI could not be performed in post-operative patients with implant fixation. Tractography was not consistent with neural status or epidural collection. The authors finally concluded that unlike trauma and spondylotic myelopathy, the epidural collection in paraplegics alters the diffusion characteristics of the cord in an unpredictable manner.

*Limitations*: Although this study is the first of its kind and gives some insight about DTI in Pott’s paraplegia, the number of patients was not large enough. The analysis of tracts by tractography was not done by objective methods hence bound to have subjective variation.

*Future*: Further studies of DTI in patients of spinal tuberculosis having myelomalacia, syringomyelia, and cord atrophy (dry lesion) especially in healed TB with residual neural deficit are needed to eliminate the possible effect of epidural collection and to find out a true correlation between neural status and diffusion parameters.

## Conflict of interest

The author(s) declare no conflict of interest.
